# Comparative efficacy and safety of antibody induction therapy for the treatment of kidney: a network meta-analysis

**DOI:** 10.18632/oncotarget.19815

**Published:** 2017-08-02

**Authors:** Mingjie Shao, Tingting Tian, Xinyan Zhu, Yingzi Ming, Yasuko Iwakiri, Shaojun Ye, Qifa Ye

**Affiliations:** ^1^ Department of Transplant Center, Xiangya 3rd Hospital, Central South University, Changsha, China; ^2^ Department of Radiology, Changsha 1st Hospital, Changsha, China; ^3^ Digestive Disease, Dongfang Hospital, Tongji University, Shanghai, China; ^4^ Department of Internal Medicine, Section of Digestive Diseases, Yale University School of Medicine, New Haven, Connecticut, USA; ^5^ Department of Transplant, Zhongnan Hospital, Wuhan University, Wuhan, China

**Keywords:** alemtuzumab, ATG, IL-2RAs, kidney transplantation, network meta-analysis

## Abstract

To evaluate the efficacy and safety of antibody induction therapies in kidney transplantation. Systematic literature searches were undertaken using MEDLINE, Embase, and Cochrane Library database from 1980 to 2016. Randomized controlled trials (RCTs) comparing three antibody induction therapies (alemtuzumab, interleukin-2 receptor antibodies and antithymocyte globulin) between each other were identified. Bayesian network meta-analysis was used to combine both the direct and indirect evidence on treatment efficacy and its safety. Antibody induction therapy studies, comprising of 18 RCTs (3444 kidney transplant recipients), were included. Overall, alemtuzumab treatment was superior to the ATG group (OR: 0.49, 95% CI: 0.32 to 0.71) and IL-2RAs group (OR: 0.36, 95% CI: 0.25 to 0.52) for reducing the 1-year acute rejection in kidney transplant recipients. Although alemtuzumab treatment was nearly same with ATG group and IL-2RAs group in improving patient survival and renal function, it can reduce the adverse effects of cytomegalovirus infection more efficiently than ATG group (OR: 0.59, 95% CI: 0.32 to 0.95) and IL-2RAs group (OR: 1.08, 95% CI: 0.61 to 1.73). Alemtuzumab was not associated with increased other adverse effects. Alemtuzumab treatment is safe and effective for kidney transplant recipients. No serious adverse effects were observed in trials or in general populations.

## INTRODUCTION

Organ transplantation has developed rapidly since the introduction of immunosuppression drugs since the 1950s. Acute rejection (AR) is the continuously serious event that impacts long-term allograft survivals [1]. During the past 15 years of renal transplantation, many new induction factors were developed to decrease the incidence and adverse effects of acute rejection. For instance, the first category agent includes the interleukin-2 receptor antibodies (IL-2RAs), such as basiliximab and daclizumab, and non-depleting monoclonal antibodies. They directly target the CD25 epitope on T cells to limit acute injection and control adverse effects, when combined with CNI-based maintenance [2–4]. The second category is the depleting polyclonal antibody antithymocyte globulin (ATG) (Thymoglobulin) containing antibodies that target a majority of peripheral blood monocytes epitopes, with preventive effect on perfusion and preservation injury and chronic rejection, and that can reverse acute rejection [5–7] The third kind, alemtuzumab (Campath-1H) named humanized anti-CD52 monoclonal antibody, depletes T cells and other lymphocytes more powerfully, thereby decreasing the dosage of immunosuppressants significantly [8–10].

It has been reported that nearly 70% of renal transplant recipients have received induction therapy with either IL-2RAs or ATG in the past decade [11]. More and more transplant centers have used alemtuzumab as an antibody induction during solid organ transplantation recently [12]. Studies of comparing the effectiveness and safety of these four agents using traditional meta-analysis have been reported [13–21]. In 2006, Morris [22] discussed alemtuzumab in solid organ transplantation in his systematic review. However, it was performed in only two randomized controlled trials (RCTs) containing few numbers of patients. W.-J. Hao [23] tested effectiveness and safety of Alemtuzumab and Daclizumab compared to ATG, and Xin Zhang [24] and Robert D. Morgan [25] did similar studies by comparing the Alemtuzumab with ATG and IL-2RAs. Although plenty of efforts have been made, the optimal agents for induction use are still unmet.

Nowadays, more and more people have used network meta-analysis to evaluate the relative effectiveness of different treatments and combine evidence across selected RCTs. As we know, direct (trials compared alemtuzumab with basiliximab or daclizumab directly) and indirect estimates (trials compared alemtuzumab with basiliximab or daclizumab via ATG group) exist in the meta-analysis. Two estimates can be combined by the network meta-analysis then a compounded effect size was calculated as the weighted mean to evaluate the direct and indirect evidence [26]. Network meta-analysis is better than traditional meta-analysis for it can predict information from some comparisons which have no clinical trial data available. In our case, the exactness of the direct estimate was improved and the width of the confidence intervals was shortened by the indirect evidence [27].

Considering the increased numbers of new trials, this manuscript intends to compare the treatment outcome of alemtuzumab with other induction drugs such as basiliximab, daclizumab and ATG in kidney transplant for all RCTs using the Bayesian network meta-analysis [28–31]. Our study will provide useful information for transplant physicians.

## RESULTS

### Characteristics of studies

**Figure 1:**
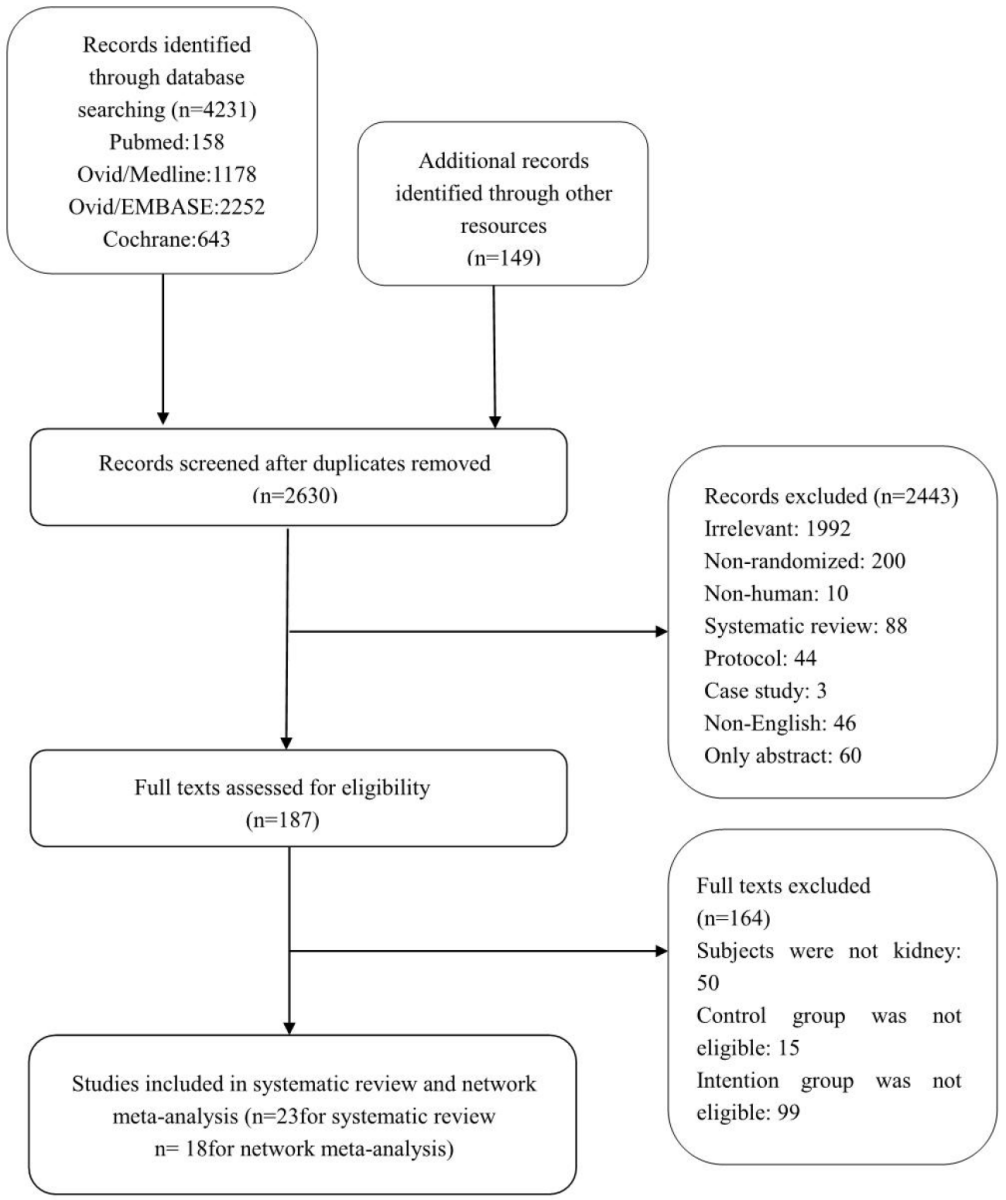
Flowchart for the selection of included trials

**Figure 2:**
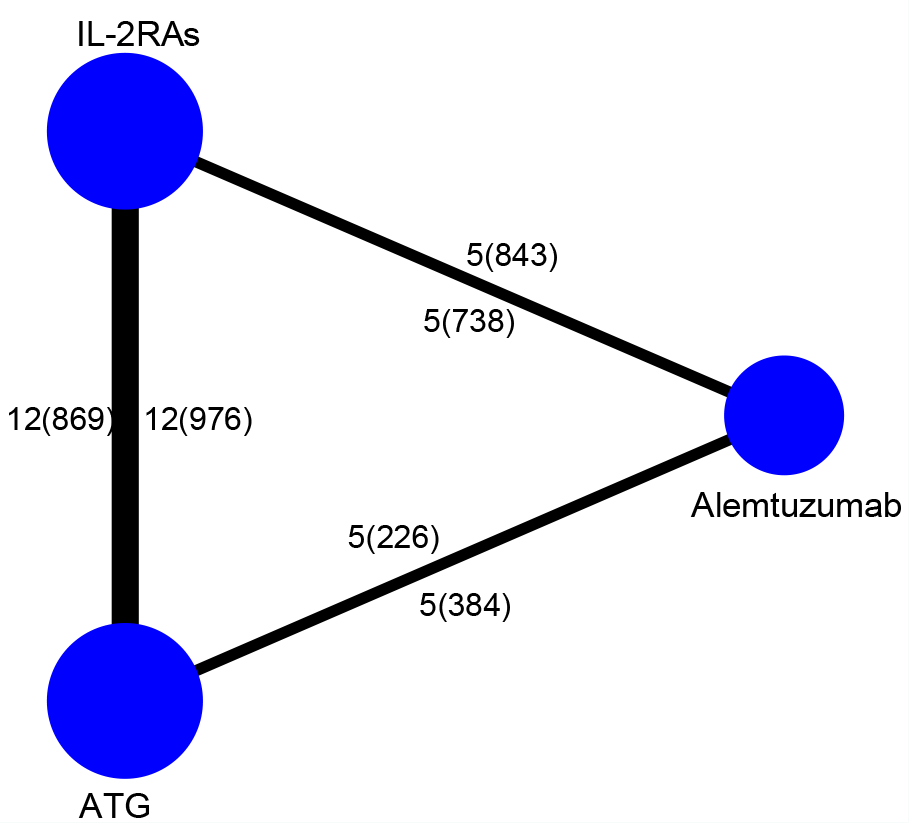
Structure of treatments and direct comparisons of network The lines between two nodes indicate that direct compared randomized trials. The weight of the lines is quantified to the number of trials used for comparison. Trials amount was presented with numbers outside while patients amount was inside the braces.

Figure 1 showed how we select the required trials in this study. In the original database and website searching we found 4380 records and then we discarded the duplicates, 2630 records were left. After 2443 records were eliminated, then 187 full-text were used for quantification. At last, 23 studies were included in this research [13–17, 19, 20, 32–47]. The details of the selected studies were listed in Table 1. Nine studies were identified as low quality (score ≤ 3), and the rest were medium quality (scored 4 or 5) which can be seen in the methodological quality assessment (Table 2). 3444 kidney transplant recipients from eighteen studies were available for network meta-analysis [14–17, 20, 32–44]. Figure 2 was the comparisons structure of the primary outcomes.

**Table 1:** Characteristics of the included studies

Study	Group	No.	Age	Gender	Dosage, duration of induction	Follow-up	Immunology risk	Donor type	Maintance immunosupressive
Matthew P WS [38] 2013	G1:Ale	58	46 ± 14	16/42	30 mg intraoperative	1 year	Low	DD, LD	Tac
	G2:Bas	58	49 ± 14	12/46	20 mg days 0, 4				Tac+MMF
The 3C group [39] 2014	G1:Ale	426	52.1 ± 13.3	277/149	30 mg after reperfusion and 24 h, age > 60 y only one dose	1 year	Low 96% High 4%	DD, LD	low Tac+MMF
	G2:Bas	426	51.8 ± 13.3	275/151	20 mg days 0, 4				Standard Tac+MMF+Pred
Kakit Chan [40] 2011	G1:Ale	82	47.3 ± 13.4	54/28	30 mg on return	2 year	High and Low	DD, LD	Tac
	G2:Dac	41	47.0 ± 10.6	27/14	2 mg/kg on return, 14 d				Tac+MMF
Alan C.Farney [14] 2009	G1:Ale	85	51 ± 12	\	30 mg intraoperative	2 year	High and Low	DD, LD	Tac/CsA+MMF+Pred
	G2:rATG	95	49 ± 13	\	1.5 mg/kg day 2, 4, 6				
Philip G.Thomas [15] 2007	G1:Ale	11	43.5 ± 4.1	6/5	30 mg pre-reperfusion	337day 127∼611day	High	DD	Tac+MMF+Pred
	G2:ATG	8	47.1 ± 4.2	2/6	1.5 mg/kg for 4 days				
Lu Tie-ming [17] 2011	G1:Ale	11	38.9 ± 4.2	5/6	15 mg before reperfusion and 24h after Tx	338day 30∼730day	High	\	Tac+MMF+Pred
	G2:ATG	11	40.8 ± 4.4	4/7	9 mg/kg before Tx				
Ramzi Abou-Ayache [20] 2008	G1:ATG	51	45 ± 12	35/16	2 mg/kg before Tx;1 mg/kg day 14	1 year	Low	DD	CsA+MMF+Pred
	G2:Dac	50	44 ± 12	36/14	1–1.5 mg/kg after Tx,day 4∼day 9				
Nicole A.Pilch [42] 2014	G1:ATG	102	52 ± 13	59/43	1.5 mg/kg day 0∼4	1 year	High 40% Low 60%	DD	Tac+MMF+Pred
	G2:IL2RA	98	49 ± 14	62/36	Dac 1 mg/kg day 0, 7; Bas 20 mg day0, 4				
Daniel C.Brenna [43] 2006	G1:ATG	141	51.3 ± 13.1	79/62	1.5 mg/kg day 0∼4	1 year	\	\	CsA+MMF+Pred
	G2:Bas	137	49.7 ± 13.0	82/55	20 mg day0,4				
Michael J.Hanaway [16] 2011	G1:ATG	69	48.5 ± 11.0	39/30	1.5 mg/kg day 0∼3	3 year	High	DD, LD	Tac+MMF+Pred 5 days later Tac+MMF
	G2:Ale	234	48.0 ± 13.0	152/82	30 mg day 0		Low and High		
	G3:Bas	171	48.9 ± 13.6	113/58	20 mg day 0, 4		Low		
Gaetano Ciancio [41] 2014	G1:ATG	43	47.8 ± 2.0	29/14	1 mg/kg/d for 7 days	95month	Most Low	DD 90; LD 38	Tac+MMF+Pred
	G2:Ale	43	47.1 ± 2.0	28/15	0.3 mg/kg day 0, 4				Low-Tac+MMF
	G3:Dac	42	49.2 ± 1.9	25/17	1 mg/kg day 0, 14, 28, 42, 56				Tac+MMF+Pred
LauriE Kyllonen [44] 2007	G1:ATG	50	47.8	14/39	9 mg/kg before reperfusion	5 year	Low	DD	CsA+Aza+Pred
	G2:Bas	50	45.5	27/31	20 mg day 0,4				
Yvon Lebranchu [45] 2002	G1:ATG	50	45.8 ± 10.8	32/18	1–1.5 mg/kg for 6–10 days	1 year	Low	DD	CsA+MMF+Pred
	G2:Bas	50	44.1 ± 11.5	36/14	20 mg day 0,4				
Christian Noel [46] 2009	G1:ATG	113	45.4 ± 10.3	52/61	1.25 mg/kg day 0–7	1 year	High	\	Tac+MMF+Pred
	G2:Dac	114	46.9 ± 9.0	59/55	1 mg/kg day 0, 14, 28, 42, 56			\	
S.G. Tullius [47] 2003	G1:ATG	62	48	35/27	9 mg/kg day 0	1 year	Low and high	\	Tac+MMF+Pred
	G2:Bas	62	48	33/29	20 mg day 0, 4			\	
Hans Sollinger [48] 2001	G1:ATG	65	49.8 ± 11.9	42/23	1.5 mg/kg day 0–14	1 year	\	DD	CsA+MMF+Pred
	G2:Bas	70	44.5 ± 13.7	37/33	20 mg day 0, 4		\	LD	
Domingo Herna´ndez [49] 2007	G1:ATG	80	47 ± 12	59/21	1–1.5 mg/kg day 0–6	2 year	\	\	CsA+Aza+Pred
	G2:Bas	80	48 ± 14	50/30	20 mg day 0, 4		\	\	Low CsA+MMF+Pred
Georges Mourad [50] 2004	G1:ATG	53	45.4 ± 12.7	32/21	1 mg/kg day 0, 1	1 year	Low	DD	CsA+MMF+Pred
	G2:Bas	52	45.3 ± 12.4	30/22	20 mg day 0, 4				
Min Jeong Kim† [51] 2008	G1:ATG	11	52 (39–68)	2/9	9 mg/kg day 0; 3 mg/kg/d for 4 day	2 year	High	DD	CsA+MMF+Pred
	G2:Dac	11	51 (34–60)	4/7	1 mg/kg day 0, 14, 28, 42, 56				
Alan Farney† [13] 2008	G1:Ale	32	\	\	30 mg intraoperative	2 year	Low and High	DD, LD	Tac/CsA+MMF+Pred
	G2:ATG	45	\	\	1.5 mg/kg day 2, 4, 6				
Gaetano Ciancio† [52] 2005	G1:ATG	30	49.3 ± 2.5	19/11	1 mg/kg/d for 7 d ays	27mo	Most Low	DD	Tac+MMF+Pred
	G2:Ale	30	50.2 ± 2.1	19/11	0.3 mg/kg day 0,4				Low-Tac+MMF
	G3:Dac	30	49.9 ± 2.4	18/12	1 mg/kg day 0, 14, 28, 42, 56				Tac+MMF+Pred
Gaetano Ciancio† [19] 2010	G1:ATG	13	44.5 ± 13.1	10/3	1 mg/kg/d for 7 days	3 year	Low	LD	Tac+MMF+Pred
	G2:Ale	13	40.0 ± 3.7	9/4	0.3 mg/kg day 0,4				Low-Tac+MMF
	G3:Dac	12	47.2 ± 2.8	7/5	1 mg/kg day 0, 14, 28, 42, 56				Tac+MMF+Pred
Karen L. Hardinger† [53] 2009	G1:ATG	141	\	\	1.5 mg/kg for 5 days	1 year	High and Low	DD	CsA+MMF+Pred
	G2:Bas	137	\	\	20 mg day 0,4				

Ale: alemtuzumab; Bas: basiliximab; Dac: daclizumab; Tx: transplantation; DD: deceased donors; LD: living donors; Tac: tacrolimus; CsA: cyclosporine; MMF: Mycophenolate Mofetil; Pred: prednisone; Aza: azathioprine.

†Only data for the whole trial is available.

**Table 2:** Methodological evaluation of included studies

Study	Randomized method	Concealment allocation	Blinding method	Follow-up	Score
Matthew P WS [38]	2	2	0	1	5
The 3C group [39]	2	1	0	1	4
Kakit Chan [40]	2	2	0	1	5
Alan C.Farney [14]	1	1	1	1	4
Philip G.Thomas [15]	1	1	0	1	3
Lu Tie-ming [17]	1	0	0	1	2
Ramzi Abou-Ayache [20]	1	0	0	1	2
Nicole A.Pilch [42]	1	0	0	1	2
Daniel C.Brenna [43]	2	2	0	1	5
Michael J.Hanaway [16]	2	2	0	1	5
Gaetano Ciancio [41]	2	1	0	1	4
LauriE Kyllonen [44]	2	2	0	1	5
Yvon Lebranchu [45]	1	1	0	1	3
Christian Noel [46]	1	1	0	1	3
S.G. Tullius [47]	1	1	0	1	3
Hans Sollinger [48]	1	1	0	1	3
Domingo Herna´ndez [49]	2	2	0	1	5
Georges Mourad [50]	1	1	0	1	3

### 1- year AR

**Figure 3:**
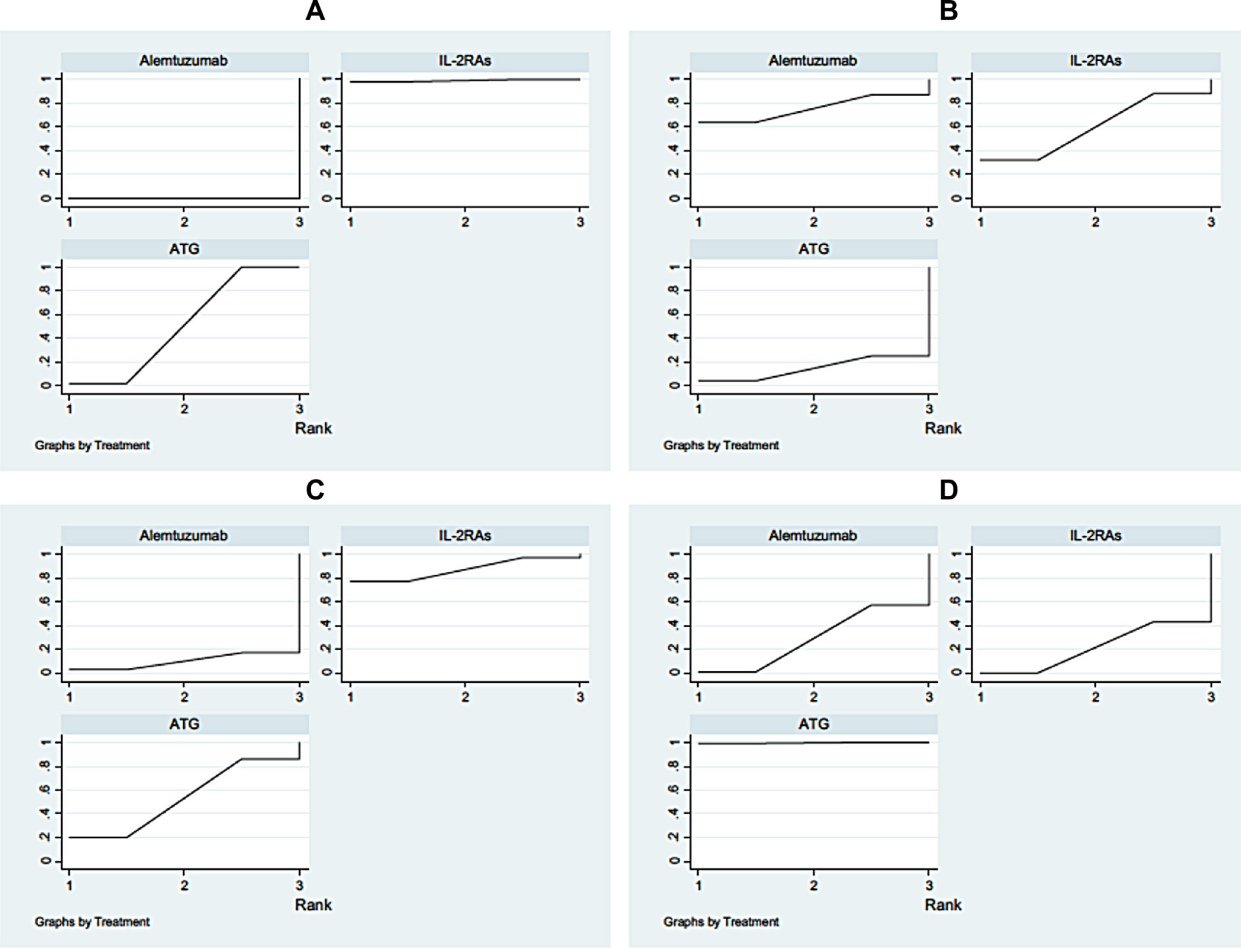
Rankings for efficacy and safety The graph displays effectiveness of each drug. X-axis shows the ranking of each drug (from the most effective to the least), Y-axis indicates the cumulative probability for each drug to be the most preferred drug. (**A**) 1 year acute rejection; (**B**) 1 year patient death; (**C**) 1 year renal function; (**D**) CMV infection.

Both network meta-analysis and traditional meta-analysis were summarized in Table 3. 1-year AR in alemtuzumab group is significantly lower compared to the ATG group (Ia, OR: 0.49, 95% CI: 0.32 to 0.71) and IL-2RAs group (IIIa, OR: 0.36, 95% CI: 0.25 to 0.52), and IL-2RAs showed a significantly higher 1-year AR compared to the ATG group (IIa, OR: 1.35, 95% CI: 1.01 to 1.78). Direct and indirect estimates were consistent in this network meta-analysis. The posterior mean residual deviance is 20.8 (20 data points) indicating adequate fit. We can see the distribution of probability of each drug in Figure 3A. Alemtuzumab is possibly to be the most preferred treatment (0%) when compared with ATG (51%) and the IL-2RAs group (99%), and the result of traditional meta-analysis was similar (Table 3). Heterogeneity among the three groups (*P*, *I*^2^) showed no significant different and publication bias also did not exist among these studies.

**Table 3:** Results of network meta-analysis and traditional meta-analysis

	Comparison	Network meta-analysis, OR/SMD* (95% CI)	Traditional meta-analysis, OR/SMD* (95% CI)	Heterogeneity (P/I2)	Publication bias (*P* value of Beggs’ test)
I	Ale vs ATG				
a	1yr AR	0.49 (0.32, 0.71)	0.46 (0.27, 0.78)	0.64/0%	0.734
b	1yr Patient death	2.29 (0.51, 6.20)	0.90 (0.36, 2.26)	0.80/0%	1.000
c	1yr Graft death	1.73 (0.50, 4.02)	0.90 (0.36, 2.26)	0.80/0%	1.000
d	Renal function	–0.18 (–0.57, 0.15)*	–0.44 (–1.12, 0.25)*	0.14/54%	0.734
e	DGF	1.36 (0.78, 2.05)	0.88 (0.52, 1.50)	0.29/20%	1.000
f	Graft loss	0.81 (0.38, 1.51)	0.57 (0.31, 1.08)	0.44/0%	0.734
g	CMV infection	0.59 (0.32, 0.95)	0.81 (0.46, 1.41)	0.32/13%	1.000
h	Malignance	0.62 (0.01, 2.46)	1.11 (0.03, 37.29)	0.05/74%	1.000
i	NODAT	0.49 (0.18, 1.08)	0.39 (0.16, 0.93)	0.32/0%	1.000
II	Anti-IL2r vs ATG				
a	1yr AR	1.35 (1.01, 1.78)	1.38 (1.07, 1.78)	0.69/0%	0.533
b	1yr Patient death	1.71 (0.70, 3.94)	1.11 (0.72, 1.71)	0.64/0%	0.108
c	1yr Graft death	1.18 (0.59, 2.29)	1.11 (0.72, 1.71)	0.64/0%	0.174
d	Renal function	–0.15 (–0.37, 0.09)*	–0.14 (–0.26, 0.01)*	0.09/47%	0.118
e	DGF	1.19 (0.86, 1.60)	1.22 (0.95, 1.56)	0.10/41%	0.754
f	Graft loss	0.93 (0.58, 1.44)	0.95 (0.67, 1.35)	0.46/0%	1.000
g	CMV infection	0.55 (0.38, 0.78)	0.53 (0.41, 0.68)	0.32/13%	0.373
h	Malignance	0.36 (0.02, 1.19)	0.62 (0.30, 1.25)	0.31/16%	0.764
i	NODAT	0.82 (0.39, 1.55)	0.78 (0.46, 1.34)	0.62/0%	0.707
III	Ale vs anti-IL2r				
a	1yr AR	0.36 (0.25, 0.52)	0.36 (0.26, 0.50)	0.55/0%	0.806
b	1yr Patient death	1.47 (0.29, 3.65)	1.53 (0.41, 5.72)	0.10/57%	0.296
c	1yr Graft death	1.54 (0.45, 3.37)	1.53 (0.41, 5.72)	0.10/57%	0.296
d	Renal function	–0.03 (–0.42, 0.29)*	0.15 (–0.03, 0.32)*	0.21/37%	0.462
e	DGF	1.15 (0.71, 1.65)	1.31 (1.01, 1.70)	0.98/0%	0.734
f	Graft loss	0.89 (0.39, 1.73)	1.28 (0.50, 3.26)	0.84/0%	0.296
g	CMV infection	1.08 (0.61, 1.73)	0.94 (0.43, 2.03)	0.07/54%	0.806
h	Malignance	2.66 (0.07, 13.56)	1.02 (0.53, 1.96)	0.56/0%	1.000
i	NODAT	0.61 (0.27, 1.21)	0.61 (0.33, 1.12)	0.81/0%	0.089

### 1- year patient death

Table 3 also indicated the results of network meta-analysis for the 1-year patient death. It showed that there was no significant difference among three groups–the alemtuzumab group and ATG group (Ib, OR: 2.29, 95% CI: 0.51 to 6.20), the IL-2RAs group and ATG group (IIIb, OR: 1.71, 95% CI: 0.70 to 3.94), the alemtuzumab group and IL-2RAs group (IIb, OR: 1.47, 95% CI: 0.29 to 3.65). Evidence of inconsistency between the direct and indirect evidence cannot be seen. The posterior mean residual deviance is 18.7 (15 data points) which means good fit to data. Distribution of probability of each induction position can be seen in Figure 3B. From the result of the rank, we found that ATG is most likely (14.5%) to be the preferred treatment for 1-year patient death, followed by IL-2RAs (60%) and the alemtuzumab group (75.5%). The traditional meta-analysis corroborated with similar results except for the comparison between the alemtuzumab group and ATG group. However, no significant heterogeneity among the three direct comparisons was found. Based on the *P* values of Beggs’ test, publication bias was not significant.

### 1- year renal function

We can see the results of network meta-analysis for 1-year renal function from the Table 3. No significant difference can be inferred between the alemtuzumab group and ATG group (Ib, SMD: –0.18, 95% CI: –0.57 to 0.15), between the IL-2RAs group and ATG group (IIId, SMD: –0.15, 95% CI: –0.37 to 0.09) and between the alemtuzumab group and IL-2RAs group (IId, SMD: –0.03, 95%CI: –0.42 to 0.29). No evidence of inconsistency between the direct and indirect evidence was displayed. A posterior mean residual deviance of 13.8 (10 data points) shows an adequate fit. Distribution of probability of each drug was illustrated in Figure 3C. We can conclude that ATG is mostly the best treatment (11%) for 1-year renal function, then alemtuzumab (69%) and the IL-2RAs group (70%) based the result of the posterior probability values. The traditional meta-analysis also showed same results. Moreover, no significant heterogeneity was found among the three direct comparisons. There was no publication bias according to the *P* values of Beggs’ test.

### Adverse effects

The harmful effects of induction mainly include delayed graft function (DGF), graft loss, cytomegalovirus (CMV) infection, malignancy, and new-onset diabetes mellitus after transplantation (NODAT). Among these five kinds of adverse effects, we only found significant difference appeared in CMV infection from Table 3 while others had no significant difference. A significantly lower CMV infection in alemtuzumab group when compared with the ATG group (Ig, OR: 0.59, 95% CI: 0.32 to 0.95), also IL-2RAs achieved a significantly lower CMV infection compared with the ATG group (IIIg, OR: 0.55, 95% CI: 0.38 to 0.78). No significant difference in alemtuzumab group was seen when compared with the IL-2RAs group (IIg, OR: 1.08, 95% CI: 0.61 to 1.73). It showed no evidence of inconsistency between the direct and indirect evidence. The posterior mean residual deviance of 23.6 (20 data points) in the model demonstrated an adequate fit. Figure 3D displayed the arrangement of a probability of each drug ranking. From the ranking, it can be inferred that IL-2RAs is most promising (21.5%) to be the best drug for CMV infection, followed by alemtuzumab (29%) and the ATG group (99.5%). The traditional meta-analysis supported this result too. However, no significant heterogeneity was found in the three direct comparisons. Based on the *P* values of Beggs’ test, publication bias was nonsignificant among different kinds of studies. What’s more, there was no significant difference among the three groups regarding the incidence of other adverse effects.

## DISCUSSION

Antibody induction therapy is accepted as an important part of acquiring the best short and long-term results after the organ transplant [48]. Since the best induction agent has not yet to be established in clinical work, most of the randomized cohort studies focusing on comparison of alemtuzumab and other single induction agents (daclizumab, basiliximab, or ATG). Most of these studies have reported similar or even better outcome from first biopsy-proven acute rejection (BPAR) incidence in the alemtuzumab group with no unfavorable effects on renal function or graft survival [14–16, 25, 34, 49–51].

Three review papers published in 2012 involving six, ten and nine randomized controlled studies respectively came to a similar conclusion that alemtuzumab induction could be better induction agent in kidney transplantation due to it reduces the risk of AR but shares the similar incidence of other efficacy outcomes (graft loss, DGF, and graft/patient death). With gaining of evidence, this network meta-analysis certified that alemtuzumab induction is the most favored induction regarding therapeutic effects without significant adverse effects.

Our study, for the first time, reviewed three kinds of induction therapy (alemtuzumab, IL-2RAs and ATG) after kidney transplantation using network meta-analysis to compile both direct and indirect evidence of therapeutic and adverse effects. In the present network meta-analysis, alemtuzumab is thought to be better than traditional induction antibodies in preventing 1year AR. Changes in rejection rates among the groups, we found, did not result in significant differences in the survival of patients or allografts. DGF and infection are still the two major post-transplant issues that have an ill effect on both qualities of life and patient/graft survival [52, 53]. This meta-analysis certifies that alemtuzumab is similar when compared with traditional antibodies for preventing both DGF and post-transplant infection. Also, it shows no significant difference in the distribution of probability observed between the group treated with IL-2RA and alemtuzumab in CMV infection. Therefore, alemtuzumab treatment is safe and effective for kidney transplant recipients.

Nevertheless, the limitations of this study should be pointed out. Since it is impossible to blind induction treatment, it has to be an open-label trial. Because of the limited number of included trials, especially the comparative group of alemtuzumab vs ATG and IL-2RAs vs ATG, there is no enough data for us to assess the effectiveness and safety of alemtuzumab in particular patient populations thoroughly, such as live versus deceased donors, or low- versus high-risk recipients. Also, our study has little power to detect 2 year or 3 year AR after transplant when the data is limited to outcomes that occur only during the first year post-transplant. Same situation also occurs in graft loss and patient survival.

## MATERIALS AND METHODS

### Literature search

We used many logic combinations of keywords and text words to search the Pubmed, Embase and Cochrane library as well as related interventions and randomized controlled trials till August 2016 (Supplementary Appendix 1). Also, we also searched the websites manually as follows: Current Controlled Trials, ClinicalTrials.gov and The World Health Organization International Clinical Trials Registry.

### Study selection

The abstracts and full texts found were checked by two researchers independently. We resolved the disagreements by discussing and consulting to another researcher. Included criteria for the papers in the analysis were: (1) randomized controlled studies; (2) kidney transplant recipients with induction (3) studies referring to at least two of the following eligible inductions: alemtuzumab, IL-2RAs and ATG; (4) studies containing the main or adverse outcomes; (5) English literature.

### Quality assessment

We used the modified Oxford score [54, 55] to evaluate the quality of methodology of included studies. Score 0 to 7 was given based on randomization, concealment allocation, blinding method and reporting of participant withdrawals.

### Outcome measures

Our study was mainly to determine the effectiveness of patients and grafts outcomes and adverse outcomes by using the alemtuzumab, IL-2RAs and ATG induction respectively. The patients and grafts outcomes contain 1-year biopsy-proven acute rejection, 1-year patient death, 1-year renal function and DGF; the adverse outcomes include CMV infections, NODAT, malignancy and graft loss.

### Statistical analysis

We compared the effectiveness of patients and grafts outcomes and adverse outcomes among three kinds of different induction therapy for kidney transplant recipients for the random effect of Bayesian network meta-analysis. The network meta-analysis allows indirect comparisons of interventions among cohorts which the meta-analysis not. In our study, we used WinBUGS (version 1.4.2, MRC Biostatistics Unit, Cambridge, UK), R (version 3.1.2, The R Foundation for Statistical Computing) and STATA (version 12.0, StataCorp, College Station, TX) to perform the Bayesian network meta-analysis. Multi-arm trials often used codes of random effect models which can be seen in http://www.mtm.uoi.gr/ (Supplementary Appendix 2). Three Markov chains ran synchronously with different initial values which were chosen at random. Each of the three sets of initial values had 50,000 simulations generated, we discard the first 10,000 simulations for the burn-in period. The median of the posterior distribution was used to calculate the pooled effect sizes. We used the 2.5% and 97.5% of the posterior distribution named corresponding 95% credible intervals same as the conventional 95% confidence intervals. For estimating the network inconsistency of every closed loop, absolute difference was calculated between the direct and indirect evidence. Loops showed statistically significant inconsistency if the lower CI extremity still far away the zero line [56]. The accuracy of the model was defined by calculating the posterior mean residual deviance. The number of data points of the model can be measured by the mean of the residual deviance, which means that the model fits the data adequately [57]. We ranked the treatments according to the degree of effectiveness based their posterior probabilities (first choice, second choice, third choice, etc.). The surface under the cumulative ranking (SUCRA) used for the measurement of probability values [58]. If the SUCRA is equal to 1 that means it is the most effective treatment. Otherwise, 0 means the most ineffective treatment.

The traditional meta-analysis was done with STATA (version 12.0, StataCorp, College Station, TX). Q statistics (*P* < 0.05 was considered heterogeneous) and *I*^2^ statistics (*I*^2^ ≥ 50% was considered heterogeneous) were used to test the heterogeneity. Begg’s tests [59] were used to evaluate the publication bias, if the *P* value was less than 0.5 then the publication bias was established.

## CONCLUSIONS

This network meta-analysis indicated that alemtuzumab, interleukin-2 receptor antibodies and antithymocyte globulin, are all effective in improving survival and renal function of the patients. Overall, however, alemtuzumab showed a greater probability of being the preferred regimen in reducing the 1-year acute rejection and a significantly lower risk of cytomegalovirus infection when compared with interleukin-2 receptor antibodies and antithymocyte globulin. Therefore, our results suggest that alemtuzumab should be considered as the recommend antibody induction in the treatment of kidney transplantation. Further random control trials (RCTs) are needed to confirm this result.

## SUPPLEMENTARY MATERIALS APPENDIX



## Abbreviations

RCTs: randomized controlled trials
IL-2RAs: interleukin-2 receptor antibodies
ATG: antithymocyte globulin
AR: acute rejection
BPAR: biopsy-proven acute rejection
DGF: delayed graft function
NODAT: new-onset diabetes mellitus after transplantation
CMV: cytomegalovirus
SUCRA: surface under the cumulative ranking
