# Supplementary material for: Comparative efficacy and safety of antibody induction therapy for the treatment of kidney: a network meta-analysis
Source: Oncotarget. 2017 Aug 2;8(39):66426–37. doi: 10.18632/oncotarget.19815 (PMC5630424; doi:10.18632/oncotarget.19815)
Supplement: Supplementary file 1 [file oncotarget-08-66426-s001.pdf]

Comparative efficacy and safety of antibody induction therapy for the treatment of kidney: a network meta-analysis

SUPPLEMENTARY MATERIALS

Supplementary Appendix 1: Search strategies for Pubmed, Ovid/MEDLINE, The Cochrane Library and Ovid/EMBASE database

Pubmed

1. Basiliximab OR daclizumab
2. rabbit antithymocyte globulin OR rATG
3. 1 AND 2
4. Alemtuzumab
5. rabbit antithymocyte globulin OR rATG
6. 4 AND 5
7. 4 and 1
8. 3 OR 6 OR 7
9. kidney transplantation OR renal transplantation
10. 8 AND 9

Ovid/MEDLINE

1. exp kidney transplantation/
2. ((kidney$ or renal$) adj (transplant$ or recipient$)).tw.
3. 1 or 2
6. randomi?ed.ab.
7. placebo.ab,tw.
8. controlled.ti,ab
9. randomly.ti,ab.
10. trial.ti,ab.
11. groups.ti,ab
12. ((randomized controlled trials) or (random$ allocation) or (double blind) or (single blind)).tw
13. ((singl$ or doubl$ or tripl$) and (mask$ or blind$)).tw
14. Or/4-13
15. interleukin-2 receptor$.tw.
16. (alemtuzumab$ or campath$).tw.
17. (basilixima$ or simulec$).tw.
18.(daclizuma$ or daclizima$ or daclizuma$ or zenapa$).tw.
19. (antithymoglobulin$ or antithymocyte$ or antilymphocyte$ or thymoglobulin$ or ATG or rATG).tw.
20. Or/15-19
22. 3 and 14 and 20

The Cochrane Library

#1. Kidney Transplantation, this term only in MeSH
#2. (kidney or renal) next transplant*
#3. (kidney or renal) near recipient*
#4. (#1 OR #2 OR #3)
#5 MeSH descriptor Antibodies, Monoclonal explode all trees
#6 MeSH descriptor Antilymphocyte Serum explode all trees
#7 monoclonal antibod* or antithymocyt* or thymocyt* or ATG or atgam or thymoglobulin or thymus anti*
#8 basiliximab or simulect or daclizumab or daclizimab or dacluzumab or daclizumab or zenapax or alemtuzumab or campath
#9 (#5 OR #6 OR #7 OR #8)
#10 (#4 AND #8)

Ovid/EMBASE

1. exp kidney transplantation/
2. ((kidney$ or renal$) adj (transplant$ or recipient$)).tw.
3. 1 or 2
4. randomi?ed.ab.
5. placebo.ab,tw.
6. controlled.ti,ab
7. randomly.ti,ab.
8. trial.ti,ab.
9. groups.ti,ab
10. ((randomized controlled trials) or (random$ allocation) or (double blind) or (single blind)).tw
11. ((singl$ or doubl$ or tripl$) and (mask$ or blind$)).tw
12. Or/4-11
13. interleukin-2 receptor$.tw.
14.(basilixima$ or simulec$).tw.
15.(daclizuma$ or daclizima$ or daclizuma$ or zenapa$).tw.
16. (alemtuzumab$ or campath$).tw.
17. (antithymoglobulin$ or antithymocyte$ or antilymphocyte$ or thymoglobulin$ or ATG or rATG).tw.
18. Or/13-17
19. 3 and 12 and 18

Supplementary Appendix 2: WinBUGS codes of random effect models for multi-arm trials

```
model {
  for(i in 1:ns) {
    w[i,1]<- 0
```

```
        delta[i,t[i,1]]<- 0
        ss[i]<- sum(n[i,1:na[i]])
        nom[i]<- sum(nom1[i,1:na[i]])
        pooled.sd[i]<- sqrt(nom[i]/(ss[i]-na[i]))
        J[i]<- 1-(3/((4*(ss[i]-na[i]))-1))
#Normal Likelihood#
        for (k in 1:na[i]) {
            y[i,k] ~ dnorm(phi[i,t[i,k]],prec[i,k])
            se[i,k]<- sd[i,k]/sqrt(n[i,k])
            var[i,k]<- se[i,k]*se[i,k]
            prec[i,k]<- 1/var[i,k]
            nom1[i,k]<- (n[i,k]-1)*sd[i,k]*sd[i,k]
            }
#Parameterization of the model#
        phi[i,t[i,1]]<- u[i]*(pooled.sd[i]/J[i])
        for (k in 2:na[i]) {
            phi[i,t[i,k]]<-  (u[i]+delta[i,t[i,k]])*(pooled.sd[i]/
            J[i])
            pdelta[i,t[i,k]] ~ dnorm(md[i,t[i,k]],taud[i,t[i,k]])
            pmd[i,t[i,k]]<- d[t[i,k]] - d[t[i,1]]  + sw[i,k]
            ptaud[i,t[i,k]]<- tau *2*(k-1)/k
            pw[i,k]<- (delta[i,t[i,k]] - d[t[i,k]] + d[t[i,1]])
            psw[i,k]<- sum(w[i,1:k-1])/(k-1)
            }
            }
#Priors#
    SD ~ dnorm(0,1)I(0,1)
    tau<- 1/pow(SD,2)
    for(k in 1:(ref-1)) {
        d[k] ~ dnorm(0,.0001)
        }
    for(k in (ref+1):nt) {
        d[k] ~ dnorm(0,.0001)
    }
    for(i in 1:ns) {
    u[i] ~ dnorm(0,.0001)
    }
#Estimated & Predicted Standardized Mean Differences#
    d[ref]<- 0
    for (c in 1:(ref-1)) {
        SMD.ref[c]<- d[c] - d[ref]
        predSMD.ref[c] ~ dnorm( SMD.ref[c],tau)
        }
        for (c in (ref+1):nt) {
        SMD.ref[c]<- d[c] - d[ref]
        predSMD.ref[c] ~ dnorm( SMD.ref[c],tau)
        }
       for (c in 1:(nt-1)) {
        for (k in (c+1):nt) {
            SMD[c,k]<- d[c] - d[k]
            predSMD[c,k] ~ dnorm(SMD[c,k],tau)
            }
            }
#Ranking of treatments#
    for(k in 1:nt) {
        order[k]<-rank(d[],k)
# this is when the outcome is positive - omit  'nt+1-' when
the outcome is negative
        most.effective[k]<-equals(order[k],1)
        for(j in 1:nt) {
            effectiveness[k,j]<- equals(order[k],j)
            }
          }
        for(k in 1:nt) {
        for(j in 1:nt) {
            cumeffectiveness[k,j]<- sum(effectiveness[k,1:j])
            }
    }
#SUCRAS#
        for(k in 1:nt) {
            SUCRA[k]<- sum(cumeffectiveness[k,1:(nt-1)]) /
(nt-1)
    }
#Fit of the Model#
    for(i in 1:ns) {
        for(k in 1:na[i]) {
            Darm[i,k]<-(y[i,k]-phi[i,t[i,k]])*(y[i,k]-
            phi[i,t[i,k]])/var[i,k]
        }
        D[i]<- sum(Darm[i,1:na[i]])
          }
        D.bar<- sum(D[])
```
